# Leukoagglutination, *Mycoplasma pneumoniae* Pneumonia, and EDTA Acid Blood

**DOI:** 10.4274/tjh.2017.0425

**Published:** 2018-03-06

**Authors:** Beuy Joob, Viroj Wiwanitkit

**Affiliations:** 1 Sanitation 1 Medical Academic Center, Bangkok, Thailand; 2 Hainan Medical University, Hainan Sheng, China; DY Patil University Faculty of Medicine, Pune, India

**Keywords:** EDTA, Leukoagglutination, Mycoplasma

## To the Editor,

We read the report “Peculiar Cold-Induced Leukoagglutination in *Mycoplasma pneumoniae* Pneumonia” with great interest [1]. Kubota et al. [1] reported an interesting patient with *Mycoplasma pneumoniae* pneumonia who had leukoagglutination. They noted that this is a rare condition. We agree that the patient had leukoagglutination and *Mycoplasma pneumoniae *pneumonia. Nevertheless, the leukoagglutination in this case may or may not have been due to *Mycoplasma pneumoniae *pneumonia. A common problem that might be forgotten is EDTA-induced leukoagglutination [2]. This basic laboratory interference phenomenon cannot be ruled out in the present case. As noted by Grob and Angelillo-Scherrer, EDTA-dependent leukoagglutination can be seen in healthy individuals and this is not related to *Mycoplasma pneumoniae* pneumonia [3].
