# Supplementary material for: Leukoagglutination, Mycoplasma pneumoniae Pneumonia, and EDTA Acid Blood
Source: Turk J Haematol. 2018 Mar 6;35(1):75–6. doi: 10.4274/tjh.2017.0425 (PMC5843780; doi:10.4274/tjh.2017.0425)
Supplement: Related texts

# Reply to the Authors:

**Authors:** Yasushi Kubota, Shinya Kimura

## To the Editor,

We thank Joob and Wiwanitkit [1] for their helpful comments regarding the cause of leukoagglutination in our case [2]. Although the underlying mechanism of in vitro leukoagglutination has not been fully clarified, leukoagglutination can be classified into two groups: (1) EDTA-dependent leukoagglutination, and (2) EDTA-independent cold-induced leukoagglutination [3,4]. As pointed out by Joob and Wiwanitkit [1], both EDTA and cold agglutinin (CA) may have been implicated in our case, although high-titer CA was detected, and numerous erythrocyte agglutinations were observed in the peripheral blood smear. 

Screening for CA has shown that low-titer CAs may be found in the serum of healthy adults [5]; this may suggest the possibility that naturally occurring CA is somewhat involved in EDTA-dependent leukoagglutination in healthy subjects. Nonetheless, when leukoagglutination occurs in an EDTA-anticoagulated blood sample, additional examination of the sample using other anticoagulants could be recommended to confirm the relationship between leukoagglutination and EDTA.

## References

[1] Joob B, Wiwanitkit V (2018). Leukoagglutination, Mycoplasma pneumoniae pneumonia and EDTA blood. Turk J Hematol.

[2] Kubota Y, Hirakawa Y, Wakayama K, Kimura S (2017). Peculiar cold-induced leukoagglutination in Mycoplasma pneumoniae pneumonia. Turk J Hematol.

[3] Goyal P, Agrawal D, Kailash J, Singh S (2014). Cold-induced pseudoneutropenia in human immunodeficiency virus infection: first case report and review of related articles. Indian J Hematol Blood Transfus.

[4] Lee JH (2017). Neutrophil aggregation on the peripheral blood smear in a patient with cold agglutinin disease. Ann Hematol.

[5] Dacie J, Dacie J (1992). Auto-immune haemolytic anaemia (AIHA): cold-antibody syndromes II: immunochemistry and specificity of the antibodies; serum complement in auto-immune haemolytic anaemia. The Haemolytic Anaemias. Vol. 3.
